# MDMA (3,4-Methylenedioxymethamphetamine) Analogues as Tools to Characterize MDMA-Like Effects: An Approach to Understand Entactogen Pharmacology

**DOI:** 10.2174/1570159X11311050007

**Published:** 2013-09

**Authors:** P. Sáez-Briones, A. Hernández

**Affiliations:** 1 Laboratory of Neuropharmacology and Behavior, School of Medicine, Faculty of Medical Sciences, University of Santiago de Chile;; 2 Laboratory of Neurobiology, Faculty of Chemistry and Biology, University of Santiago de Chile

**Keywords:** Psychotropics, Ecstasy, Entactogens, MDMA analogues, Behavior, monoaminergic neurotransmission.

## Abstract

Besides stimulants and hallucinogens, whose psychotropic effects are shared by many structurally related molecules exhibiting different efficacies and potencies in humans, the phenylisopropylamine MDMA (3,4-methylenedioxymethamphetamine, XTC, “Ecstasy”) is the prototypical representative of a separate class of psychotropic substance, able to elicit the so-called entactogenic syndrome in healthy humans. This reversible altered state of consciousness, usually described as an “open mind state”, may have relevant therapeutic applications, both in psychotherapy and as a pharmacological support in many neuropsychiatric disorders with a high rate of treatment failure. Nevertheless, a comprehensive and systematic exploration of the structure-activity relationships associated with entactogenic activity has remained incomplete and controversial, highlighting the possibility that MDMA might represent a pharmacological rarity in the field of psychotropics. As the latter is still an open question, the pharmacological characterization of MDMA analogues remains the logical strategy to attempt the elucidation of the structural requirements needed to elicit typical MDMA-like effects. Intriguingly, almost no experimental evidence supports the existence of actual MDMA analogues that truly resemble the whole pharmacological profile of MDMA, probably due to its complex (and partially not fully understood) mechanism of action that includes a disruption of monoaminergic neurotransmission. The present review presents a brief summary of the pharmacology of MDMA, followed by the evidence accumulated over the years regarding the characterization of classical structurally related MDMA analogues in different models and how this state of the art highlights the need to develop new and better MDMA analogues.

## INTRODUCTION

**Fig. (1):**
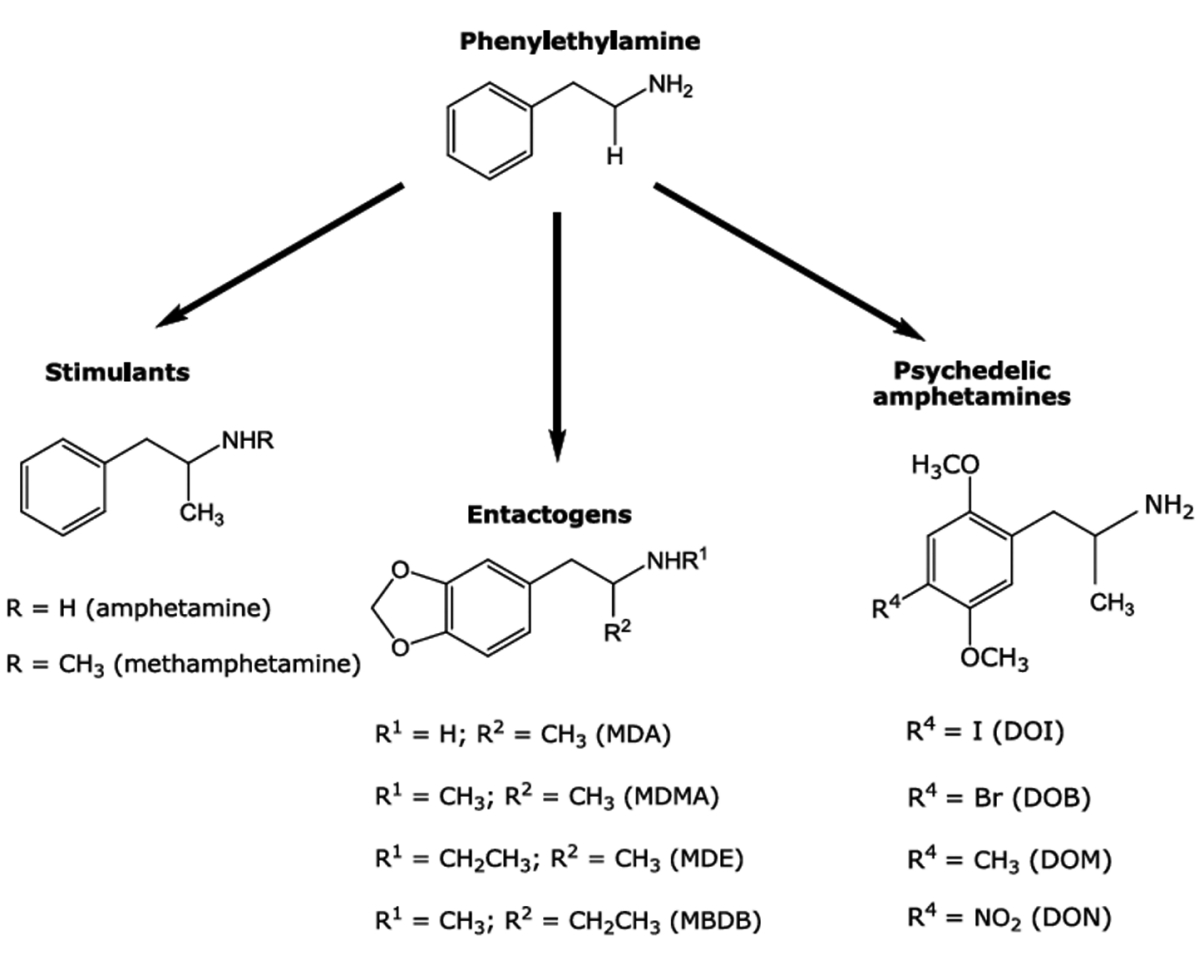
Chemical structures of MDMA, classical MDMA analogues and structurally related stimulants and hallucinogens.

MDMA (3,4-methylenedioxymethamphetamine, XTC, “Ecstasy”) is a popular monoaminergic psychotropic substance capable of inducing a reversible controlled alteration of consciousness in humans characterized by emotional relaxation, feelings of happiness and empathy to other persons that has been called the “entactogenic syndrome” (see below [25]). Because of these unique pharmacological properties, that are different from those elicited by structurally related stimulants and hallucinogens (Fig. **1**), and also due to its legal status as a drug of abuse, MDMA has been intensively studied in order to establish mainly its potential risks and also to some extent its clinical benefits. Indeed, whereas an impressive amount of experimental data obtained using animal models and drug consumers has accumulated showing that chronic exposure to MDMA may elicit anxiogenic effects [1] and disruptions of cognitive processes [2] (but also see [96]), as well as long lasting alterations in the control mechanisms of mood, other evidence suggest that the acute administration of MDMA might be a valuable tool not only in psychotherapy but also in the handling of human neuropsychiatric disorders with a high rate of treatment failure [3]. Unfortunately, a systematic characterization and refinement of the structural requirements associated with the occurrence of entactogenic activity by means of rational modifications of the MDMA template has been shown to be particularly complex, mostly because of the apparent condition of MDMA as a pharmacological “rarity”, that is, a psychotropic substance possessing unique pharmacological properties that seem not to be shared with other structurally related drugs. For this reason, the search for actual MDMA analogues still remains as the most proper strategy to study the molecular basis of typical MDMA-like effects. 

## MDMA: Neither a Hallucinogen nor a Stimulant

**Fig. (2):**
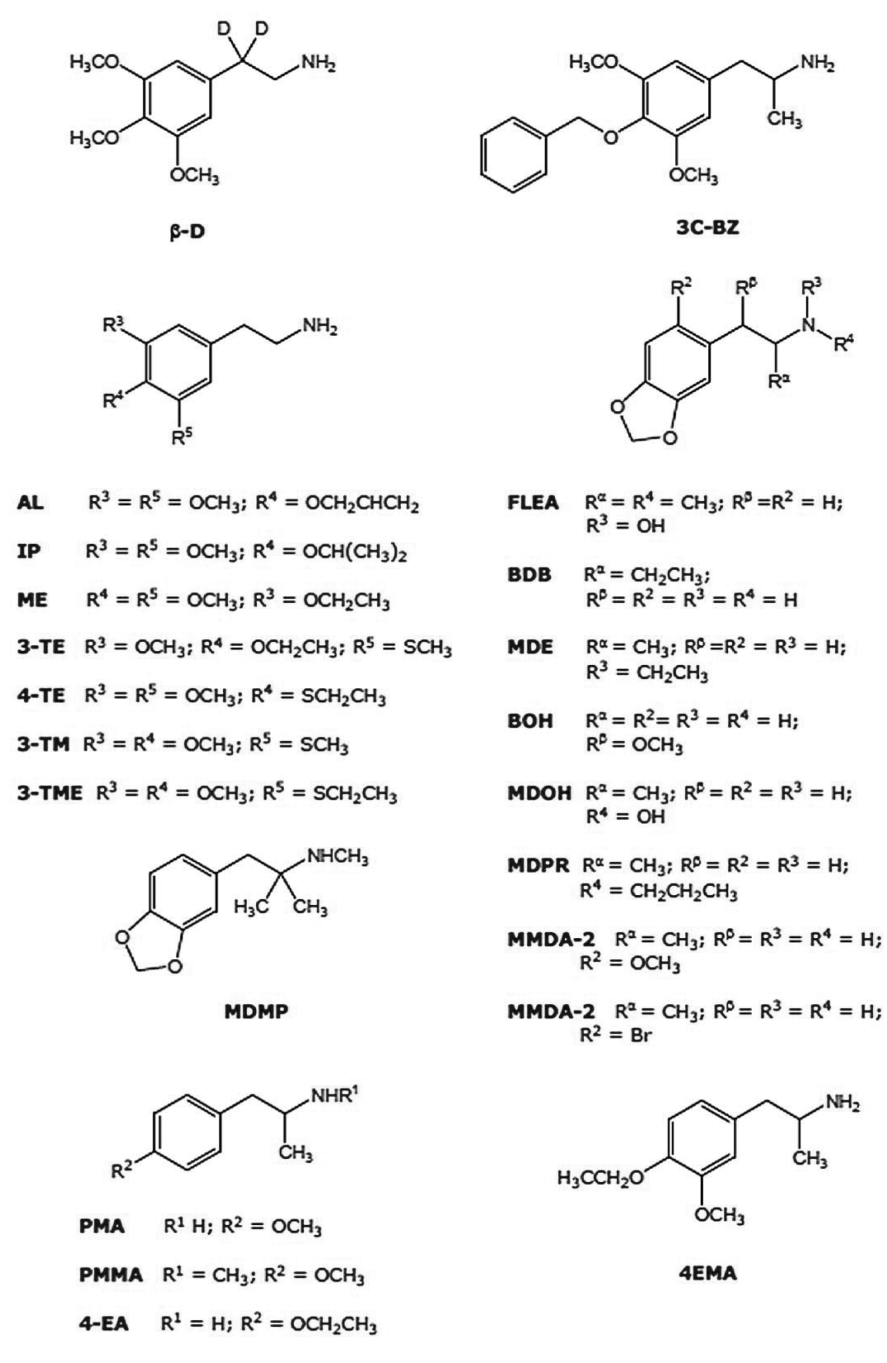
Chemical structures of a selection of classical phenylalkylamines described as possessing potential MDMA-like properties, as described in [121].

More than two decades ago, D.E. Nichols proposed the term “entactogen” to describe a putative new class of monoaminergic phenylisopropylamines, after analyzing his results obtained for the three monoaminergic phenylisopropylamines MDMA, MBDB (N-methyl-1,3-benzodioxolbutanamine) and MDE (3,4-methylenedio-xyethylamphetamine) in a drug discrimination paradigm, an experimental approach extensively used for the behavioral characterization of classical hallucinogens such as LSD (lysergic acid diethylamide) and 2,5-dimethoxyamphetamine (e.g. DOI, DOB, Fig. **1**) derivatives. This new term makes a strong reference to the possible psychotherapeutic usefulness of these substances [4,5] and comprises a subjective syndrome in healthy humans, which can be described as an “open mind” state characterized by an emphasis on heightened self-acceptance and openness for communication, together with a decrease of fear responses [3] and without typical psychedelic-like effects. MDMA, originally patented by Merck in 1912 as a (apparently) minor precursor of an appetite suppressant never developed [6-9], is the prototypical entactogen and it has been used as a recreational drug for decades [10]. However, the position of MDMA within the range of the chemically related psychotropic drugs is rather uncertain. Initially, neither brain damage nor neurotoxicity was found, suggesting that this drug could possess a rather harmless clinical pharmacological profile [9]. Indeed, MDMA has been administered to humans under controlled conditions and shown to be useful in psychotherapy [3, 12-17]. According to these reports, MDMA is able to help overcome strong defenses and to confront the patient with deep conflicts by reducing anxiety [18], leading to success in “therapy-resistant” cases. These findings support the notion that this drug may act as an adjunct to psychotherapy in modern psychiatry [14]. In addition, MDMA has been postulated to be useful in the development of more efficacious pharmacological handling of neuropsychiatric disorders with a high rate of failure such as depression [19, 20], post-traumatic stress disorder [21-23], autism [24], and even substance abuse [25]. Nevertheless, as for almost every population of individuals, caution is recommended with MDMA use in some neuropsychiatric patients, probably because of their increased susceptibility to acute and/or chronic abreactions to the drug [13, 26]. Among these, MDMA may induce in susceptible individuals persistent acute toxic hyperthermia, an effect that might be fatal as a result of primary renal failure [27] and is also sensitive to sex differences [28]. Such toxicity is shared with other drugs such as cocaine, paramethoxyamphetamine (PMA) and methamphetamine (MA) in rodents and rhesus macaques [29, 30, Figs. **1**, **2**]. It is difficult to predict it in humans, because susceptible users are unusually sensitive to small variations in dose. Nevertheless, due to its status of “most popular street drug” and also because it remains placed on Schedule I in the U.S.A. since 1985 (followed by similar decisions enacted in many other countries) as a drug deemed to have no medical uses and a high potential for abuse, research has been focused during the last decades in constructing a detailed pharmacological profile of MDMA based on its behavioral and toxic effects [31-33]. Experimental evidence suggests also that MDMA may cause occasionally long-lasting effects, which are described by some users as “midweek blues” [34]. In addition, some frequent MDMA users also suffer long-lasting effects on working memory, planning ability and executive control, together with cognitive impulsivity [35, 36], aggression, anger and even depression in polydrug users [37, 38]. In contrast, ex-users who had abstained from the drug for at least 6 months are reported not to differ from non-users in their cognitive capacities [39]. Moreover, results obtained in a comparative study between abstinent and non-abstinent polydrug users indicated no substantial cognitive dysfunction associated with MDMA intake [40]. In agreement with these findings, a report comparing former MDMA users, polydrug users who had never taken MDMA and control subjects indicated no differences in serotonergic neuron integrity between the three groups, as reflected by binding measurements on the serotonin transporter (SERT) using positron emission tomography [41]. This particularly relevant aspect of MDMA pharmacology remains still a matter of debate (see below), as predictions about the actual effects of MDMA on cognition should result certainly from a complex interaction of dose level, drug intake frequency and individual susceptibility.

Although its mechanism of action is not fully understood, MDMA (and possibly MDMA-like drugs as well) is known to exert its acute psychotropic effects acting mainly as a non-classical SERT substrate [42, 43] that induces non-exocytotic serotonin (5-HT) release by triggering a reversal of the normal transporter flux [44, 45]. This effect on SERT is considered essential to induce both its acute and long-term effects. Indeed, MDMA displays high affinity for rodent SERT, whereas its affinities for other monoamine trans-porters are lower [46, 47]. Nevertheless, it should be noted that the affinity rank order for MDMA at human SERT is slightly different, with a higher affinity for the noradrenaline transporter (NET), reflecting extended physiological links among monoamine transporters [48]. Certainly, its indirect mechanism of action upon serotonergic neurotransmission distinguishes MDMA from classical hallucinogens and stimulants, which need to interact directly with central serotonergic 5-HT_2A/2C_ receptors or to activate indirect dopaminergic mechanisms, respectively, to exert their effects *in vivo *[49-51]. Nevertheless, acute administration of MDMA in rats may also cause a blockade of 5-HT re-uptake, inhibition of monoamine oxidase A, tryptophan hydroxylase and loss of SERT availability [52]. This may lead to 5-HT depletion, a potentially toxic effect that could persist for up to one year in the rat. These changes seem to be more intense in the striatum, hippocampus and prefrontal cortex [31]. The latter does not match with other reports where neither brain damage nor neurotoxicity was observed, suggesting that this drug possesses a rather harmless clinical pharmacological profile [11-15].

Despite the inaccuracy of some reviews where MDMA is described as a stimulant similar to amphetamine or methamphetamine [53], not supported by reliable evidence about dependence associated with its pharmacological profile [54], MDMA shares with other structurally related amphetamines the feature of eliciting its psychotropic effects by altering monoaminergic neurotransmission, but in a complex way. In contrast to structurally related hallucinogens [55, 56], MDMA possesses only low micromolar affinities and modest efficacy for central serotonergic 5-HT_2A/2C_ receptors [46]. However, the availability of these receptors is reduced in frequent MDMA users, as shown more than a decade ago by single photon emission computed tomography [57, 58], an effect that was first found in rats [59] and seems to occur in combination with a desensitization of 5-HT_1A_, 5HT_2A_ and 5-HT_2C_ receptors [60]. In addition, rats exposed to regular doses of MDMA during adolescence, exhibited enhanced typical spontaneous behavioral (head and wet-dog shakes, back muscle contractions) and endocrine (prolactin and corticosterone secretion) effects induced by the hallucinogen DOI [61] (Fig. **1**), indicating that MDMA seems to induce a sensitization to DOI, but it cannot induce a hallucinogenic syndrome *per se*. 

Despite of the latter, typical MDMA-like effects depend on central serotonergic receptor function. Early pharmacological evidence demonstrated a direct relationship between the activation of 5-HT_2_ receptors and classical locomotor MDMA-like effects [62], as well as for MDMA induced hyperthermia [63] and neurotoxicity [64]. Besides, serotonin was also early demonstrated to be involved in the modulation of dopamine function *via *5-HT_2_ receptors [65]. This evidence has been confirmed by more recent data indicating that 5-HT_2A/2C_ receptors play a role in the activation of dopaminergic neurotransmission [66, 67]. In agreement with the latter, additional early studies demonstrated that the blockade of 5-HT_2_ receptors disrupt MDMA-like effects by affecting the functional link between serotonin and dopamine, expressed as diminished dopamine levels [68-70]. Recently, an additional regulatory effect related to dopaminergic reward systems, associated with an induced reinforcement and cue-induced reinstatement of a possible MDMA seeking behavior through 5-HT_2A_ receptors in mice has also been described [71]. Accordingly, it has been reported that MDMA enhances extracellular striatal dopamine and striatal/prefrontal cortex serotonin in DAT-KO mice, and that MDMA-mediated serotonin release seems to be exerted at DAT in SERT-KO mice [72]. In addition, hyperlocomotion induced by MDMA in rodents, originally described as dopaminergic [62, 73] and linked to a stereo-selective D1-mediated ERK pathway downstream [74, 75], is mediated also by 5-HT_1A/1B_ receptors [31] and seems to be also dependent on cortical serotonin, supporting the notion that motor behaviors elicited by MDMA may result upon activation of dopamine and serotonin neurotransmission pathways regulated in a region and modality-specific manner [76]. The serotonin-dopamine link extends also to MDMA neurotoxicity [77], an assumption that was later confirmed as mesolimbic dopamine release induced after chronic exposure to MDMA has shown to be mediated by serotonergic 5-HT_1B_ receptors [78]. 

Besides typical psychotropic and neurotoxic effects, MDMA has been described to induce severe cognitive impairment in humans [79, 80]. These effects can also be found in rats after chronic drug treatment [81], but there is also evidence of its occurrence regardless of the dose regimen [82]. Although the current knowledge is limited, consistent evidence has been published supporting a specific role of norepinephrine neurotransmission in the events precipitating cognitive impairment. For instance, executive function impairment induced by MDMA in primates may be blocked by the NET inhibitor desipramine and the SERT inhibitor citalopram but not by the DAT/SERT inhibitor methylphenidate [83]. These data support the notion that the interaction between MDMA and NET should also be considered as part of the whole mechanism of action of the drug, as the affinity exhibited by MDMA for this monoamine transporter in humans has been shown to be higher than those determined for SERT and DAT [48]. Indeed, MDMA acts as a NET substrate promoting norepinephrine release [84]. In addition, the stimulant-like effects elicited by MDMA have been also reported as mediated by NET since the NET inhibitor reboxetine is able to attenuate them in humans [85]. These results were rather unexpected because it is widely assumed that NET blockade should affect stimulated interpersonal sensitivity and ratings of anxiety only, whereas subjective effects such as “drug high” and “closeness” are supposed to be dependent on dopamine and serotonin neurotransmission, respectively [86]. Other recent evidence have shown that some other typical MDMA-like effects such as hyperlocomotion is also mediated by norepinephrine, since pretreatment of rats with the α_1_-receptor antagonist prazosin totally abolished MDMA-mediated locomotor response, pointing to a relevant noradrenergic component in this spontaneous psychomotor response elicited after systemic administration of MDMA [87]. In addition, α_1_-adrenergic receptors are involved also in the classical MDMA-mediated hyperthermia [88]. Taken together, the pharmacological evidence currently available regarding MDMA is consistent with a mechanism of action associated with functional interactions between serotonin, dopamine and norepinephrine neurotransmission pathways occurring mostly at a presynaptic level. The latter implies a particularly complex scenario where the role for each monoamine system becomes not evident.

Classical rat *in vivo* paradigms (e.g. locomotion, rearing, grooming, head shakes, anxiolytic-anxiogenic responses, active avoidance conditioning) and drug discrimination in rodents have been used for a long time for the pharma-cological characterization of psychoactive drugs. In rats, acute doses of MDMA enhance locomotion in a dose-dependent manner, together with a decrease in the number of head shakes, an effect usually favored by hallucinogens [31]. In contrast, repetitive MDMA administration induces an anxiety-related behavior [31, 89, 90] and a 3-day exposure to toxic doses of MDMA may cause a classical 5-HT syndrome [91], although the apparent direct link between high toxic levels of MDMA and long-term changes in impulsivity in the rat remain controversial [92]. In addition, available data suggest that MDMA may exert passive and active avoidance learning impairment in rats, possibly by inhibition of training-associated increases in the N-methyl-D-aspartate receptor subunit NR1 and Ca^2+^/calmodulin-dependent protein kinase [93]. The cognitive impairments induced by MDMA may also be independent from the drug intake regimen and particularly relevant during the perinatal period, as demonstrated recently in rats using the water maze assay [94, 95]. In agreement with the latter, various studies carried out to evaluate the long-lasting effects of MDMA on memory and learning in humans reported alterations in working memory, planning ability, executive control, together with cognitive impulsivity (for specific references, see Morton [35]). Nevertheless, these reports have been challenged recently by the study of Halpern *et al*. who failed to demonstrate cognitive impairment in MDMA users with minimal exposure to other drugs [96]. Besides memory impairment, MDMA is described to be neurotoxic in experimental animals, but the toxic dose was found to be highly strain-dependent [31]. Some years ago, an extensive study of the effects of long-term 5-HT depletion was carried out in Dark Agouti rats. It was shown that consistent decreases of 5-HT and 5-hydroxyindoleacetic acid were induced in the hippocampus, cortex and striatum. Only frequent doses of MDMA were able to produce neurotoxic damage. These data may be relevant for frequent MDMA consumers, considering that neurotoxicity in rats can be achieved following doses that are only a fraction of those used in earlier studies [97] and taking into account the comparisons between human and animal doses [98, 99]. Nevertheless, these calculations are controversial, because of the difficulty to express reliably both pharmacokinetic and metabolic factors associated with MDMA ingestion in different species [100].

It is evident that the controversy about the pharma-cological profile of MDMA as a psychotropic drug remains still in debate. In agreement with the latter, and in spite of its controlled status, research on the psychotherapeutic potential of MDMA has continued off and on [101] and studies were published reporting the evaluation of this drug as an adjunct to the therapy of post-traumatic stress disorder [14-17, 23], anxiety [14, 18] and depression [19, 20]. Taken together, the uncertainty about MDMA [102] and the current knowledge of the benefits of the psychopharmacology of MDMA and of the potential harmful effects associated with its use justifies a search for analogues sharing its desirable therapeutic features but lacking most of its reported risks and/or unwanted side effects.

## MDMA Analogues and their MDMA-Like Properties

MDA (Fig. **1**) is probably the most popular analogue and the principal metabolic product of MDMA, as described earlier (revised in Green *et al*. [31]). This drug was first synthesized in 1910 and is believed to share some pharma-cological properties with both classical phenylalkylamine hallucinogens and entactogens, as part of a rather complex profile* in vivo *[103]. At the very beginning of research on this molecule, it was found that MDA produced sympathomimetic effects and a marked central stimulation. Some years later, its entactogenic properties (increased self-awareness and enhanced emphathy), together with elevated sensory perception were described. Due to its similar properties to those of MDMA, MDA was also proposed as a psychotherapeutic tool [104].

Using drug discrimination studies, in which rats are trained to distinguish between a drug and saline, it was demonstrated that only (*R*)(-)-MDA produces a “hallucinogenic” cue [4, 105, 106]. This correlates well with the higher affinity of (*R*)(-)-MDA at 5-HT_2A_ receptors [107]. Moreover, MDA stereoisomers seem rather to produce a dual stimulus effect with one response or the other predominating [103]. In cells expressing 5-HT_2A_ and 5HT_2C_ receptors, it was found that MDA induces a more efficacious, isomer-specific, concentration-dependent increase in the hydrolysis of phosphotidylinositol (PI) at 5-HT_2A_ receptors, compared to MDMA. At 5-HT_2C_ receptors, both isomers of MDA were equipotent in inducing PI hydrolysis, whereas (*R*)(-)-MDMAwas markedly less efficacious. In this paper, the authors discuss the role of 5-HT_2_ receptor affinity in the mechanism of action of MDA and MDMA, based on the finding that both substances may possess stereoselective intrinsic activity at 5-HT_2A_ and 5-HT_2C_ receptors and may act as partial agonists [108]. Because of the rather low affinity of MDA and MDMA at 5-HT_2A/2C_ receptors and the absence of correlation between the 5-HT peak measured by micro-dialysis after a high dose of MDMA and locomotor activation, hyperthermia and hormone secretion [109-111], it was speculated that a link might exist between 5HT_2A/2C_ receptor activation and neurotoxicity [108], which should also be induced by chronic administration of MDA to rats [112]. This line of evidence has been cited over the years, but its pharmacological relevance remains unclear.

Various MDMA and MDA analogues have been tested in different experimental models to determine if they share some of the pharmacological properties of both structural templates. In an interesting old study, the exploration of the behavioral properties of a series of methoxylated phenylisopropylamines in order to determine the effect of other substitution patterns and the relative importance of individual methoxy groups was attempted using a drug discrimination task and DOM (Fig. **1**) as stimulus drug. It was found that generalization did not occur with the dimethoxylated amphetamine (DMA) derivatives 2,3-DMA, 2,6-DMA, and 3,5-DMA, whereas it did with standard classical di- and trimethoxylated amphetamines [113]. In a second study, 2,3-MDA and 3,4-MDA were tested in a two-lever drug discrimination trial in rats. The authors reported that only 3,4-MDA was able to induce generalization to DOM or amphetamine, whereas 2,3-MDA only generalized to 3,4-MDA [106]. More recently, a group of four ring-monomethylated derivatives of MDA were evaluated for their hallucinogenic-like and entactogenic-like behavioral effects in the rat, and the accumulation of [^3^H]5-HT and [^3^H]dopamine in whole brain synaptosomal preparations were measured. The results obtained indicated that two of them, 2-methyl-MDA and 5-methyl-MDA, exhibited high potency and selectivity as serotonin-releasing agents, although they cannot be classified as “pure” entactogens *in vivo *[103, 104, 113]. Additionally, EDA (ethylenedio-xyamphetamine) has been demonstrated to be nearly equipotent to MDA in its ability to induce [^3^H]5-HT and [^3^H]dopamine release from rat hippocampal slices, whereas IDA (isopropylidenedioxyamphetamine) was considerably less potent [114]. In drug discrimination experiments, complete substitution for LSD and MDMA was found for EDA and IDA, which also correlates in the latter case with [^125^I]DOI displacement. In contrast, MDE (Fig. **1**), which is believed to be less neurotoxic than MDMA in animal models [115, 116], whereas MDOH (Fig. **2**) may possess a non-amphetamine-like profile, even more distinct than that of MDMA itself [117]. In another study, where rats were trained to discriminate DOM or (+)-amphetamine from saline, the racemic mixtures and the optical isomers of MDA, MDMA, MDE and MDOH were compared. The DOM stimulus did not generalize to any of these drugs, whereas only (*S*)(+)-MDMA, (±)-MDE, (*S*)(+)-*N*-ethylamphetamine and (±)-*N*-hydroxyamphetamine generalized to amphetamine, indicating that entactogenic activity is probably stereo-selective [118]. In a similar protocol, (+)-MBDB generalized to MDMA and the parent drug, 3,4-MDA. All three drugs exhibited a similar stereoselectivity, the (+)-isomer having greater potency than the (-)-isomer. By contrast, the hallucinogens, (+)-LSD, DOM and mescaline and the psychostimulants (+)-amphetamine and (+)-methamphetamine did not substitute for (+)-MBDB. The results again supported the hypothesis that the primary behavioral activity of MDMA-like compounds is unlike to that of hallucinogens and stimulants and may represent the effects of a novel drug class. Evidence that presynaptic serotonergic, but not dopaminergic, mechanisms are critical, was shown. Finally, 5,6-methylenedioxy-2-aminoindan, a non-neurotoxic rigid analogue of MDA that was previously found to substitute for MDMA but not for (+)-LSD, was found to substitute completely for (+)-MBDB. The *N*-methyl derivative 5,6-methylenedioxy-2-methylaminoindan produced similar results. The authors propose that this demonstration of entactogen-like discriminative stimulus properties, for drugs devoid of neuronal degenerative toxicity potential, may serve as reliable evidence of the independent mechanisms for these effects in rats [119]. Additionally, extending the alkyl group on the nitrogen or α-carbon of MDA reduces the ability of these compounds to induce dopamine release but also produces long-lasting 5-HT depletion in the rat brain, as revealed by microdialysis for MDMA and MDE [120]. 

So far, among the MDMA and MDA derivatives tested, only MDE and MBDB have been reported to exert entactogenic-like effects [121] and have been evaluated in humans under controlled conditions [51, 122, 123]. Interestingly, the stimulus effects of three sulfur-containing psychoactive phenylalkylamines including the putative entactogen 2C-T7 (2-(2,5-dimethoxy-4-*n*-propylthiophenyl)-1-aminoethane) were studied in rats. It was demonstrated that, in contrast to reasonable interpretations of the reports available regarding this drug, 2C-T7 seems to be best classified as a DOM-like hallucinogen, whereas 4-MTA (1-(4-methylthiophenyl)-2-aminopropane), PMA and PMMA (Fig. **2**) may be considered as MDMA-like molecules, as confirmed by recreational consumers and rat behavioral studies. Indeed, PMA is a weak central stimulant compared to amphetamine, whereas methamphetamine is as potent as the latter drug [124-127]. The hybrid molecule PMMA lacks stimulant properties in mice, and its pharmacological profile may be rather closer to that of an entactogen-like substance [128]. In a series of experiments using PMMA as a discriminative stimulus, it was demonstrated that (*S*)(+)-PMMA was able to completely generalize to (±)-PMMA, suggesting that the PMMA stimulus may be stereoespecific [129], as in the case of MDMA [128]. Additionally, a comparative study of the behavioral properties of the optical isomers of PMMA, MBDB, MDA and MDMA indicated that (±)-PMMA generalized to (*S*)(+)-MBDB, (*R*)(-)-MBDB, (*S*)(+)-3,4-DMA, (*R*)(-)-3,4 DMA, (*S*)(+)-MDMA and (±)-MDMA in rats. In addition, it was suggested also that MBDB and 3,4-DMA are probably closer to PMMA [130]. Interestingly (*R*)(-)-PMA, like MDMA, seems also to share an entactogenic-like profile [101]. 

## The Social Interaction Test to Study Pro-Social Effects

As described earlier, MDMA induces in humans an altered state of consciousness characterized by increased empathy to others [3]. Consequently, this syndrome reinforces social situations [131-133]. Interestingly, MDMA is able to enhance social interaction in rats as well [134] and to decrease aggression in mice and fish [135, 136]. These effects seem to be mediated by alterations in 5-HT neuro-transmission [137]. In rats, MDMA doses ranging 2.5 to 5 mg/kg elevates “adjacent lying”, a specific passive physical contact parameter measured in the social interaction model [138-140]. This effect is further increased when ambient temperature is higher, suggesting that it is not simply an adaptation to a cold environment [141]. This pro-social effect should be linked to serotonergic 5-HT_1A_ receptor activation mediated by the massive 5-HT release in the hypothalamus induced by MDMA at acute doses. The latter causes the release of the neuropeptide oxcytocin that, in turn, should be the direct effector of the pro-social behaviors induced by MDMA [140, 142-144]. Modifications of oxytocin release patterns are proposed to be limited to a specific structure network in the central nervous system, including the medial preoptic area, the nucleus accumbens, medial amygdala, ventromedial hypothalamus, as well as hypothalamic oxytocin containing neurons [140, 147]. More recently, another study showed that the prosocial effects mediated by MDMA are also associated with increments in the expression of Fos transcription factor in the same brain regions [145, 146]. In addition, the prosocial effect induced by MDMA can be attenuated and even replaced by an anxiogenic-like syndrome (mediated by serotonergic 5-HT_2A_ receptor activation), probably induced by 5-HT depletion that may arise after repetitive drug exposure [148]. To the best or our knowledge, although the social interaction test has been used for more than two decades [149], no reports about the effects of MDMA analogues on social behavior have been published yet. 

## New MDMA-Like Drugs

Interestingly, despite of the detailed but ambiguous descriptions of the subjective effects in humans of at least 40 synthetic psychotropic phenylalkylamines which remain underinvestigated [121], a careful analysis of the effects in humans of each of them indicate the existence of a selected group of fourteen MDA, MDMA and mescaline derivatives that might be considered as MDMA-like analogues, that is, drugs whose effects reported in humans can converge to the effects evoked by MDMA and MDA [11] and include the already known and partially characterized MDMA and MDA derivatives (Fig. **2**).

## MDMA Analogues and Bioisosteres

**Fig. (3):**
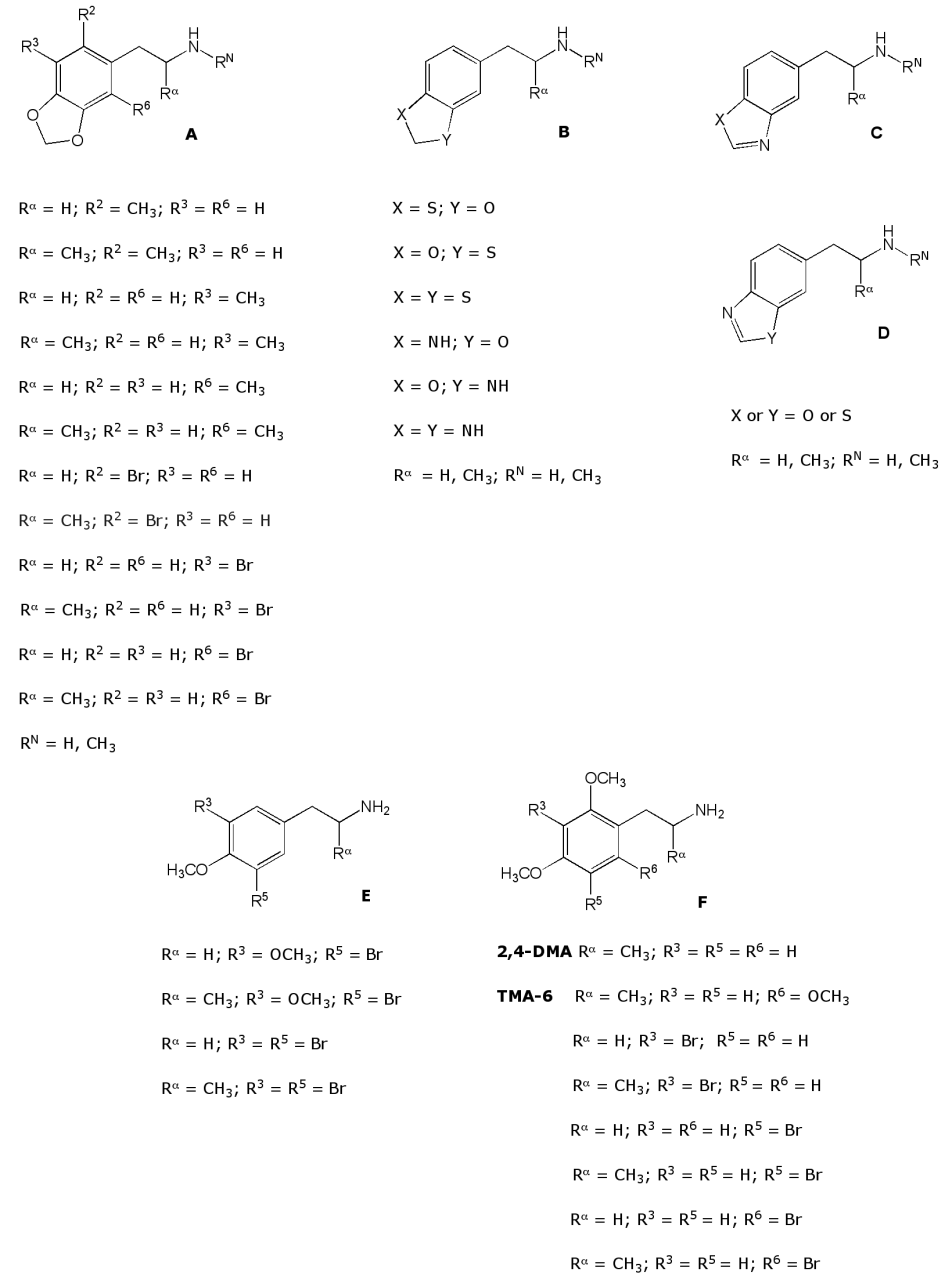
Chemical structures of different MDMA and non-MDMA analogues of potential interest: brominated MDMA and MDA analogues (A), bioisosteres (B, C, D) and mescalinoids (E, F).

Variations on the MDMA structure for pharmacological purposes have been limited to the modification of the aminoalkyl side chain by replacing the α- and the *N*-methyl by an α-(MBDB) or *N*-ethyl group (MDE) or by including it in a 2-aminoindan ring structure (MMAI) [4]. In addition, a brominated analogue of MDA has been tested in human volunteers. Its activity (400 mg) was described as “amphetamine-like” as an alternative to “MDA-like” [150]. Parker *et al. *[104] found that 1-(2-methyl-3,4-methylenedioxyphenyl)- and 1-(3-methyl-4,5-methylenedio-xyphenyl)-2-aminopropane are not only fairly potent 5-HT releasers in rats but also substitute, at low doses, for the entactogen-like MBDB and MMAI in the drug discrimination paradigm. Their isomer 1-(2-methyl-3,4-methylenedioxyphenyl)-2-aminopropane (the methyl isostere of 1-(2-bromo-3,4-methylenedioxyphenyl)-2-aminopropane) is four times less potent as compared to MMAI and only substitutes partially for MBDB. These results suggest that rational modifications on the benzene ring of MDA (or presumably also MDMA) can lead to potentially new molecules that might share some of the special pharma-cological properties of these compounds. For instance 1-(2-bromo-3,4-methylenedioxy- and 1-(3-bromo-4,5-methylene-dioxyphenyl)-2-aminopropane, bioisosteric with the more potent Parker compounds, could be expected to exhibit similar, or possibly entactogen-like properties (Fig. **3**). Bioisosteric replacement of oxygen by sulfur has been shown to increase potency in a number of cases that bear some structural analogy to the molecules (Fig. **3B**, [121]). A single such modification has been introduced in the dioxole ring of MMDA-2 (Fig. **2**), and it seems reasonable to extend this concept based on the MDMA structure. As an extension of the latter, the study of isoxazoles and their dihydro derivatives (Figs. **3C** and **3D**) might be possible suitable templates for the development of novel MDMA-like molecules.

## Mescalinoids

Psychoactive amphetamines were all derived from the α-phenylethylamine mescaline, a naturally occurring hallucinogen contained in the cactus *Lophophora willliamsii* (for a review on mescaline, see Kelsey [151]). At the beginning of the research on hallucinogens, the phenylisopropylamine analogue of mescaline, TMA (3,4,5-trimethoxyamphetamine) was found be to up to 3 times more potent than mescaline in humans. Moreover, the homologation of any of the currently known phenylethylamines to their corresponding amphetamine analogues was found to increase dramatically the *in vivo *potency [121]. For this reason, the pharmacological characterization of mescaline analogues (mescalinoids) has not been extensively attempted and has remained rather restricted to their hallucinogenic action [152-154] or to the differences between hallucinogens and entactogens *in vivo*, where mescaline is included in the hallucinogenic category [155]. Moreover, none of the already characterized mescaline analogues has been included in the Designer Drugs Directory [156], so they are not considered “street drugs” and their individual subjective effects have not been investigated systematically. Interestingly, the subjective reports of the effects in humans of a number of them support the possibility that they might share some of the qualities that characterize MDMA [121].

The monothio analogues of the mono-, di-, and triethoxy homologues of mescaline have been evaluated in man. Modifications at the ring position *para* to the ethylamine chain, either with a sulfur atom, a longer alkyl chain, or both, lead to compounds with potent central nervous system activity. The 4-*n*-propoxy and 4-*n*-butoxy homologues and their corresponding 4-thio analogues were also synthesized and pharmacologically evaluated. The propyl homologues retained high potency, but a butyl group (either with or without a sulfur atom) leads to a decrease in activity. The *m*-ethyl or *m*-thio analogues retained some effect but the diethoxy and especially the triethoxy homologues seem to be inactive as psychotomimetic drugs [157]. 

## Future Directions

MDMA seems to possess peculiar pharmacological properties that cannot be resembled easily by standard medicinal chemistry approaches. Moreover, MDMA-like molecules were not actually found, highlighting the need to search for more and effective experimental approaches in order to answer the central question about MDMA as a “unique” psychotropic drug. One possible explanation for the lack of success looking for true MDMA analogues might reside in the fact that most of the key events associated with the pharmacological effects of MDMA remain still not fully understood. In particular, the complex links between serotonin, dopamine and norepinephrine taking place at both the presynaptic and postsynaptic level seem to be critical to reach full MDMA-like activity. As in the case of behavioral approaches, exhaustive designed physiological profiles of these interactions are required to further comparison with possible, rationally designed MDMA analogues.

Besides the latter, the development of new analogues and bioisosteres starting from the basic phenylalkylamine moiety is based on the fact that rather subtle structural modifications of the basic structure of the phenylethylamine template may induce dramatic changes in the ability of the molecule to evoke stimulant, hallucinogenic and/or entactogenic effects: for instance, although an additional methyl group or bromine atom may not necessarily generate major electronic disruptions inside the molecule, they may enhance its lipophilicity compared to MDMA and consequently should enhance brain penetrability. Such modifications have been related to the ability of these drugs to act as 5-HT releasers *in vitro*, probably because the presence of a methyl group may allow the molecule to adopt a favorable conformation to interact with SERT. Similar modifications at specific positions on the aromatic ring (e.g. R^6^, see Fig. **3**) do not favor hallucinogenic or entactogenic-like activity [104]. One may propose that an isosteric replacement of the methyl group by a sterically undemanding, weakly electronegative bromine should induce another type of modification in the ability of the molecule to interact with SERT, hopefully favoring entactogenic activity in the rat. Similar effects are expected by isosteric replacement of the oxygen atom contained in the typical methylenedioxy moiety of MDMA by sulfur or nitrogen, although these replacements may not necessarily lead to compounds sharing the same pharmacological features. Certainly, for reliable predictions about possible true similarities with MDMA, interactions with SERT, DAT and NET, together with *in vitro* approaches evaluating monoamine- releasing effects must be attempted and compared with those of MDMA.

Additionally, the comparison of the behavioral profiles in animals referred to prototypical amphetamines representing entactogens (MDMA), and structurally related stimulants and/or hallucinogens through an appropriate choice of a series of animal behavioral paradigms instead of the evaluation of single behaviors might be a reliable strategy to find MDMA-like molecules [158] as an extension of early conclusions published for animal studies of hallucinogenic activity [159]. Surprisingly, publications regarding the construction of comparative behavioral profiles for MDMA-like compounds are scanty. A first study [111] showed that MDMA and MDE should induce psychomotor effects compatible with a mixture between a stimulant and a hallucinogen, supporting the old hypothesis that entactogenic activity may arise as a result of a combination of stimulant and hallucinogenic effects. This assumption is not consistent with the effects induced by MDMA (or even MDE) [121]. In spite of the latter, it should be noted that neither a pure stimulant nor a hallucinogen was included for comparison in this study. Additionally, an interesting and impressive paper published by Hegadoren *et al. *[160] showed for the first time the comparative characterization of spontaneous psychomotor behaviors in the rat induced by MDMA and some related analogues. Here, 30 different behaviors were evaluated in rats at a single equimolar dose of MDMA, PMA and amphetamine, as well as MDA and MDE. Unfortunately, the choice of the reference drugs was not appropriate to ensure a clear distinction between stimulants and hallucinogens. Variations in the central levels of 5-HT were also measured, supporting the notion that classical behavioral paradigms associated with psychomotor activity in the rat are associated with a variation of central 5-HT and dopamine [161]. 

The unique locomotor activity pattern induced by MDMA in rodents seems to be strongly dependent on the differential activation of central dopaminergic D1, D2 and D3 receptors [162, 163]. In addition, head-shakes are known to be dramatically enhanced by classical hallucinogens such as DOI, an effect that is blocked after co-administration of a serotonergic 5-HT_2A_ receptor antagonist [164, 165]. Our research group has constructed behavioral profiles of the acute effects of MDMA, MA and DOI in rats, using a combination of spontaneous psychomotor responses, elevated plus-maze measurements and active avoidance conditioning responses for further evaluation of possible MDMA-like analogues. This methodology can be reliably applied to place accurately any MDMA analogue among the large group of psychotropic phenylalkylamines [166]. In addition, pro-social effects using the social interaction model with MDMA as reference could be a proper complementary experimental approach to evaluate and characterize classical and non-classical MDMA-like molecules. Some preliminary results for MDMA, MDA and MDE has been already reported, indicating that not even MDA seems to fully resemble the pro-social effects elicited by MDMA [167].

Another almost unexplored possibility to find new entactogenic-like drugs is the search for new possible MDMA-like structural templates. One intriguing possibility is a systematic exploration of mescalinoids. Here, the encouraging subjective reports in humans for 2,4-DMA and 2,4,6-trimethoxyphenylisopropylamine (TMA-6) are consistent with MDMA-like effects [121]. Consequently, the behavioral characterization of a selection of new mescaline and TMA analogues substituted at R^3^ and/or R^5^ with bromine (Fig. **3E**) may lead to compounds sharing at least some of the *in vivo* effects elicited by MDMA. The latter justifies the pharmacological characterization of those types of compounds, as well as a selected group of mescaline-like brominated analogues (Fig. **3F**). In this regard, preliminary data for 12 brominated MDMA analogues and a selection of mescalinoids indicate that these molecules exhibit restricted orientations in the binding site at rSERT and hSERT, retaining a similar affinity compared to MDMA [168-172]. Corresponding preliminary behavioral results obtained for a selection of compounds indicated that at least bromination does not promote typical MDMA-like effects [173], including a disruption of the social interaction test response [174].

Besides the latter, based on data obtained for low doses of MDMA indicating differential effects on spatial memory and operant learning, our research group has started the characterization of the effects of one single high dose of MDMA in neocortical plasticity using an *in vivo* long-term potentiation (LTP) assay. Preliminary results show that MDMA almost duplicate prefrontal cortex LTP, whereas spatial memory is disrupted [175]. As MDMA has been proved to enhance acquisition at the active avoidance conditioning response model, the latter functional data prompted us to extend our experiments to evaluate possible modulatory effects on cognitive processes elicited by rational structural modifications of the MDMA template. Finally, a separate measurement of the effects on *in vivo* LTP at key brain locations (prefrontal cortex, amygdala and hippocampus) may offer some hints about the actual central effects associated with operative cognition after MDMA intake. As already mentioned, the available data regarding possible disruptive effects on fear acquisition processes are controversial and incomplete. In particular, the operative relationship between prefrontal cortex and amygdala activities should be addressed comparing the effects of MDMA at both locations with those elicited by the compounds proposed. Considering that MDMA is believed to be non-anxiogenic but it enhances acquisition (at least under the experimental conditions previously used [166]) and promotes prefrontal cortex LTP in rats, one should expect similar combined profiles for those compounds postulated to exhibit MDMA-like properties. 

Regardless of the obvious assumption that the pharmacological characterization of psychotropic drugs requires an integrated approach combining *in vivo*, *in vitro* and *in silico* methodologies, it seems that the particularly peculiar pharmacological nature of MDMA makes the use of such a multidisciplinary strategy much more critical, in order to answer the complex question about the biological basis of the “altered state of consciousness” elicited (apparently only) by MDMA. However, the limitations of the animal models currently available to address the complex pharmacology of MDMA to predict the occurrence of entactogenic effects in humans must be kept in mind, as human MDMA-like effects are expected to be always dependent upon individual on set (mental state) and setting (physical and social environment) influences besides purely structural (theoretical) considerations of the drug interacting with a single and/or several molecular targets.

## ABBREVIATIONS

2C-T-7: = 2-(2,5-dimethoxy-4-*n*-propylthiophenyl)-1-aminoethane
4-MTA: = 1-(4-methylthiophenyl)-2-aminopropane
5-HT: = serotonin
DAT: = dopamine transporter
DOI: = 2,5-dimethoxy-5-iodo-phenylisopropylamine
DOM: = 2,5-dimethoxy-4-methylamphetamine
EDA: = ethylenedioxyamphetamine
hSERT: = human SERT
IDA: = isopropylidenedioxyamphetamine
LSD: = lysergic acid diethylamide
MA: = methamphetamine
MBDB: = 2-methylamino-1-(3,4-methylenedioxyphenyl) butane
MBDB: = N-methyl-1,3-benzodioxolbutanamine
MDE: = 3,4-methylenedioxyethylamphetamine
MDMA: = 3,4-methylenedioxymethamphetamine
NET: = noradrenaline transporter
PI: = phosphotidylinositol
PMA: = paramethoxyamphetamine
PMMA: = *N*-methyl-1-(4-methoxyphenyl)-2-aminopropane
SERT: = serotonin transporter
TMA: = 3,4,5-trimethoxyamphetamine
